# External Quality Assessment Program for Next-Generation Sequencing-Based HIV Drug Resistance Testing: Logistical Considerations

**DOI:** 10.3390/v12050556

**Published:** 2020-05-18

**Authors:** Hezhao Ji, Neil Parkin, Feng Gao, Thomas Denny, Cheryl Jennings, Paul Sandstrom, Rami Kantor

**Affiliations:** 1 National HIV and Retrovirology Laboratories at JC Wilt Infectious Diseases Research Centre, Public Health Agency of Canada, Winnipeg, MB R3E 3R2, Canada; paul.sandstrom@canada.ca; 2 Department of Medical Microbiology and Infectious Diseases, University of Manitoba, Winnipeg, MB R3E 0J9, Canada; 3 Data First Consulting Inc., Sebastopol, CA 95472, USA; nparkin34@gmail.com; 4 Duke Human Vaccine Institute and Department of Medicine, Duke University Medical Center, Durham, NC 27710, USA; feng.gao@duke.edu (F.G.); thomas.denny@duke.edu (T.D.); 5 Department of Molecular Pathogens and Immunity, Rush University, Chicago, IL 60612, USA; cheryl_jennings@rush.edu; 6 Division of Infectious Diseases, Brown University Alpert Medical School, Providence, RI 02906, USA; rami_kantor@brown.edu

**Keywords:** HIV, drug resistance, next generation sequencing, external quality assessment

## Abstract

Next-generation sequencing (NGS) is likely to become the new standard method for HIV drug resistance (HIVDR) genotyping. Despite the significant advances in the development of wet-lab protocols and bioinformatic data processing pipelines, one often-missing critical component of an NGS HIVDR assay for clinical use is external quality assessment (EQA). EQA is essential for ensuring assay consistency and laboratory competency in performing routine biomedical assays, and the rollout of NGS HIVDR tests in clinical practice will require an EQA. In September 2019, the 2^nd^ International Symposium on NGS HIVDR was held in Winnipeg, Canada. It convened a multidisciplinary panel of experts, including research scientists, clinicians, bioinformaticians, laboratory biologists, biostatisticians, and EQA experts. A themed discussion was conducted on EQA strategies towards such assays during the symposium. This article describes the logistical challenges identified and summarizes the opinions and recommendations derived from these discussions, which may inform the development of an inaugural EQA program for NGS HIVDR in the near future.

## 1. Introduction

External quality assessment (EQA), also referred to as an external quality assessment scheme (EQAS), plays a vital role in assuring that a laboratory performs biomedical assays competently [1]. EQA is defined as a system for objectively verifying performance using an external agency or facility [2], relying on interlaboratory or inter-site comparisons. It facilitates the identification of areas that need improvement, the determination of potential training needs, and the evaluation and monitoring of training impact. An EQA is often administered by a third-party agency for ensuring the consistency of a laboratory in performing specific assays of interest or by regulatory agencies for accreditation purposes. For instance, the ISO 15189-accredited medical laboratories and the Clinical Laboratory Improvement Amendments (CLIA)-certified clinical laboratories in the US are all required to participate in relevant EQA programs for consistent test quality and demonstrated laboratory competence [3].

Conventional EQA relies on three main approaches: proficiency testing (PT), rechecking/retesting, and on-site evaluation [2]. By leveraging their unique advantages, each of these approaches plays an important role in EQA for varied biomedical assays. PT is, by all means, the most commonly applied EQA method, especially when established reference materials are readily available [2]. The Clinical and Laboratory Standards Institute (CLSI) defines PT as “A program in which multiple samples are periodically sent to members of a group of laboratories for analysis and/or identification; whereby each laboratory’s results are compared with those of other laboratories in the group and/or with an assigned value, and reported to the participating laboratories and others” [4]. The ISO/IEC uses a different term, proficiency testing schemes (PTS), which is defined as “inter-laboratory comparisons that are organized regularly to assess the performance of analytical laboratories and the competence of the analytical personnel” [5]. While minor difference exists among these terms, PT, PTS, EQA, and EQAS are often used interchangeably [4,6].

## 2. EQA for Sanger Sequencing-Based HIVDR Testing

For decades, Sanger sequencing (SS) has been applied as the standard HIV drug resistance (HIVDR) genotyping method for research, surveillance, and patient care purposes [7]. Several EQA programs designed for SS HIVDR assays have been applied worldwide by different regulatory agencies or assay quality assessment groups [8,9,10,11,12]. Among them is the Virology Quality Assurance (VQA) program, funded through the US National Institute of Allergy and Infectious Diseases (NIAID) [8]. The VQA program provides comprehensive quality assessment for assays targeting HIV used in NIAID-supported clinical trials under the AIDS Clinical Trials Group (ACTG). Its functionality was expanded later to provide resources and EQA support to any laboratory doing virological testing for any NIH-sponsored study or program. The NIAID VQA program has been in operation since 1988. It plays a vital role in ensuring the validity and inter- and intra-laboratory comparability of HIV virology data generated for NIH-sponsored studies [8,13]. In 2001, the VQA launched its EQA program for SS-based HIVDR assays [13], which was later adopted by the World Health Organization (WHO) Global HIV Resistance Network (HIVResNet) as a mandatory requirement for membership in its HIVDR laboratory network [8].

The VQA HIVDR panels include HIV-positive plasma from donors with HIV or virus stocks derived from the expansion of HIV positive specimens in cell culture, diluted in a plasma matrix. PT specimens are first characterized for viral load (VL) and HIV DR-associated mutations (DRMs) using commercially available assays. The five-specimen panels are then distributed to the designated client laboratories, where they are genotyped for HIVDR using commercial and/or in-house-developed SS assays. All laboratory data derived from such tests are then submitted and compiled. A well-defined scoring algorithm is then used to compare the sequences from different laboratories to a group consensus, and the EQA assessment results are then communicated to the laboratories and relevant requesting agencies directly [8,14]. Similar EQA programs designed for SS HIVDR assays also exist in different regions or countries around the world such as TREAT Asia Quality Assessment Scheme (TAQAS) in Asia, Quality Control for Molecular Diagnostics (QCMD) HIVDR proficiency program in Europe, and Japanese External Quality Assessment Program to Standardize HIV genotyping (JEQS) in Japan [9,11,12]. All such programs played an essential role in establishing SS as the routine method for HIVDR genotyping across the world.

## 3. EQA, a Challenging but Essential Component in the Generalized Implementation of Next Generation Sequencing HIVDR Testing

While SS has been considered the “gold standard” for HIVDR genotyping, its intrinsic limitations, such as inconsistent detection of minority resistance variants (MRVs), necessitate the development of new HIVDR tests with improved sensitivity [15,16,17]. A next-generation sequencing (NGS)-based HIVDR test enables quantitative, sensitive detection of low abundance nucleotide and amino acid variations. Moreover, it also allows simultaneous analyses of multiple specimens with unprecedented high data throughput in multiplexed runs, rendering improved time-efficiency and cost-effectiveness when conducting batched sample testing [7,18,19,20]. With the increasing affordability of equipment and consumables, NGS HIVDR assays are being adopted by more laboratories worldwide and may soon become the new standard for HIVDR genotyping.

**Figure 1:**
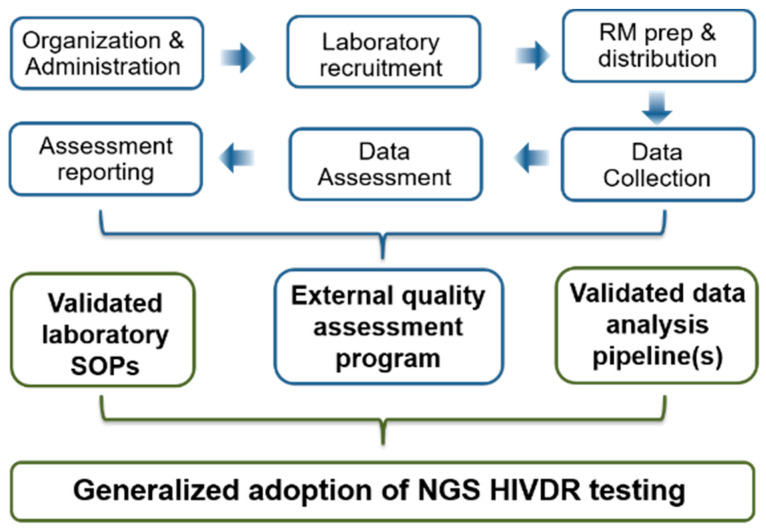
Essential requirements for the generalized adoption of NGS HIVDR assays and the key logistical components for a supportive EQA program. (Abbreviations: RM: Reference Materials; SOPs: Standard Operating Procedures; NGS: Next-Generation Sequencing; HIVDR: HIV Drug Resistance).

While it is well-appreciated that the generalized adoption of NGS HIVDR assays requires fully validated, standard operating procedures (SOPs) for sample processing and a sophisticated bioinformatics pipeline for effective data analysis, one essential but still missing aspect of ensuring assay consistency and reliability is an EQA program that functions (Figure 1). NGS HIVDR assays are multiprocedural and involve many potential “check-points”, where artificial biases or significant variations may arise and subsequently compromise the accuracy and reliability of the final output. Therefore, an EQA is at least as critical for laboratories performing NGS HIVDR assays, as it is for other clinical laboratory assays. While EQA programs for SS HIVDR have been widely applied, innovative EQA strategies have yet to be established for NGS HIVDR due to the fundamental differences between SS- and NGS-based assays and the data they generate [21,22,23]. For instance, conventional SS assays generate a single sequence per specimen, and DRMs are qualitatively detected and reported as being present (sometimes in mixtures) or absent. The EQA strategies for such tests are based on similarity analysis of the sequences and the concordance of DRM detection from individual laboratories against the consensus from the combined group [13,24] (Table 1). In contrast, NGS HIVDR assays differ from SS in many ways; EQA strategies developed for SS assays may not be applicable for NGS HIVDR assays [21]. With such challenges being recognized, themed discussions were carried out on EQA strategies for NGS HIVDR during the 2^nd^ International Symposium on NGS HIVDR held in Winnipeg, Canada, in September 2019. This article summarizes the proceedings from the discussions specifically on the logistical challenges and considerations for establishing an EQA program for NGS HIVDR assays.

## 4. EQA Strategies for NGS HIVDR Assays: Logistic Challenges and Considerations

Recognizably, EQA for NGS HIVDR testing is a new field for which limited knowledge and experience are available currently, and extensive research and development efforts are still required. An operational EQAP that executes such EQA functionalities has yet to be established. Most of the research efforts in this regard have been devoted to the development of effective data assessment and scoring criteria for the evaluation of laboratory competence in performing such assays [21,22,24]. However, the establishment of such an EQAP requires a comprehensive effort of administerial management, financial operation, PT support, data management, and subsequent reporting and follow-up actions.

Like EQAPs for other biomedical assays, the operation of an EQAP for NGS HIVDR testing may be divided into six main task areas, including (1) organization and administration, (2) laboratory recruitment, (3) reference material preparation and distribution, (4) data collection, (5) data assessment, and (6) EQA reporting (Figure 1). Accordingly, Table 1 summarizes the major logistical challenges one may encounter within each of these areas and some general issues applicable for any operational program (listed as “other challenges”), the successful experiences from EQA for SS HIVDR testing in addressing such challenges (taking the NIAID VQA program as an example), and the suggested considerations and recommendations for the establishment and operation of an EQAP for laboratories conducting NGS HIVDR assays. Based on the experiences from a pilot study that evaluated the potential of using existing VQA PT specimens for NGS HIVDR EQA [23,29], and comparing the performance of different bioinformatics pipelines [22], some strategies that may facilitate a smooth transition from a SS- to a NGS-based HIVDR testing era are also advised (Table 1).

It is noteworthy that, while the NIAID VQA program is taken as an exemplar EQAP for SS-based HIVDR assays in Table 1, most of the NGS HIVDR considerations and recommendations based on the VQA experience should be applicable or adaptable for other alike EQAPs such as TAQAS, QCMD, JEQS, or existing similar programs.

## 5. Conclusions

As an exemplar “disruptive” technology, NGS can revolutionize the conventional SS-based HIVDR genotyping practice and can enable sensitive and quantitative MRV detection. Many commercial and in-house-developed NGS HIVDR assays have been developed together with sophisticated bioinformatics pipelines. Meanwhile, the gradual cost reductions for both NGS instruments and related consumables have converted NGS from a high-end research tool into an affordable and accessible technology for general HIVDR laboratories. NGS may soon become the new standard for HIVDR testing in research and surveillance, as well as clinical monitoring purposes. Therefore, appropriate EQAPs will become imperative for ensuring the quality of data from the laboratories performing such assays. Due to the uniqueness of NGS HIVDR assays and the complexity of data derived from such tests, the existing EQA strategies and EQAPs targeting SS-based HIVDR genotyping are not optimal for these new assays. Technical and logistical challenges involved in the development and implementation of NGS-specific EQAPs remain to be resolved and require additional research.

## Figures and Tables

**Table 1:** NGS EQA for HIV drug resistance testing: logistical challenges and considerations.

EQA Tasks	Logistical Issues	Sanger Experiences (VQA as an Example)	NGS HIVDR EQA Considerations and Recommendations
**Organization and Administration**	**Who organizes/operates?**	▪ NIAID/NIAID VQA contractor institute.	▪ VQA fits well in undertaking this task; however, extra funding support may be required, and the number of participating laboratories may be limited for operational reasons. ▪ When possible, joint efforts between VQA and regional/national/global quality assurance programs or agencies are recommended for managerial and financial considerations. ▪ Collaborative data assessment between VQA and partner(s) with relevant NGS bioinformatics expertise would be beneficial.
	**Who participates?**	▪ NIH-funded network laboratories and programs. ▪ WHO-designated HIVDR laboratories. ▪ Other laboratories approved by NIAID VQA contracting officer.	▪ Laboratories from the NIAID clinical trial networks and with appropriate NGS capacity would potentially be early adopters for NGS HIVDR technologies. ▪ HIVDR laboratories from the current VQA program, WHO HIVResNet, and PHAC/PAHO collaborative network showed interest in participating in a NGS EQA. ▪ Gradual expansion is foreseeable while NGS HIVDR is adopted more broadly worldwide.
	**Who funds?**	▪ NIAID VQA contract supports PT panel distribution, data collection, and assessment for EQA purposes. ▪ Costs related to specimen processing and data submission are self-funded by the client laboratories.	▪ Adding a NGS HIVDR component into existing HIVDR EQA programs would be preferable. However, extra funding support may be required for VQA or other existing EQA programs to cover NGS HIVDR testing. ▪ Commercial non-network laboratories could be self-funded, for cost recovery purposes. ▪ Funding from collaborating regional/national quality assurance programs or agencies could be sought when possible.
**Laboratory Recruitment**	**Recruitment strategies**	▪ Required participation for NIH-funded network laboratories. ▪ Part of WHO designation criteria for HIVDR reference laboratories. ▪ Initial on-site evaluation or auditing is required for all WHO lab designation.	▪ Inclusion of NIH-supported and WHO HIVResNet designated laboratories with validated NGS systems. ▪ NGS accreditation for participating laboratories with satisfying performance, which may be incorporated into the updated CLSI or other alike standards for clinical NGS HIVDR testing. ▪ Acceptance of self-funded, voluntary participation of new laboratories when it is feasible.
	**Basic infrastructure requirements**	▪ Availability of laboratory facility and equipment required for SS-based HIVDR genotyping.	▪ Availability of NGS sequencing equipment and accessories required for HIVDR genotyping. ▪ Availability of instruments for RNA/DNA quality assessment and quantification, which are required for NGS wet laboratory procedures, test quality control, and troubleshooting uses. ▪ An on-site evaluation might be required for official accrediting applications.
	**Sample processing capacity requirement**	▪ Commercial or in-house SS-based HIVDR assay(s) in place. ▪ Experienced staff for SS HIVDR sample processing and data management.	▪ Commercial or in-house NGS-based HIVDR assay in place. ▪ Experienced staff is available for HIV sample processing and NGS sequencing.
	**Bioinformatics capacity requirement**	▪ Availability of software and expertise required for SS sequence data processing, HIVDR interpretation, and reporting.	▪ Availability of expertise and steady access to bioinformatics pipeline(s) for NGS data processing, HIVDR interpretation, and reporting. If using a pipeline validated for HIVDR applications, specialized bioinformatics support may not be required.
**Reference Materials prep and distribution: Wet Panels** [21]	**Panel design**	VQA panels contain five specimens, designed with the following factors: ▪ Approximating viral diversity in clinical HIV specimens. ▪ Representing specimens at varied viral loads (VLs). ▪ Consisting of varied HIV-1 subtypes. ▪ Harboring DRMs in protease (PR), reverse transcriptase (RT) and integrase (IN) coding regions. ▪ Covering unusual HIV DRMs as possible.	▪ Include all factors considered in the VQA SS HIVDR panel design. ▪ Inclusion of specimens with HIV DRMs at varied known frequencies (especially in the range of 5~20%). ▪ Inclusion of pedigreed standards and controls for monitoring systemic error rates. ▪ A two-step panel development strategy is advised to facilitate SS to NGS HIVDR transition: Step 1: Assessment Panels (APs): The existing VQA or similar panels may serve this need with no specific modification or extensive characterization required. The subsequent data assessment could be based on NGS consensus sequences, and the current SS-based EQA strategies would apply. Step 2: Validation Panels (VPs): Well-characterized, comprehensive wet panels with ground truth on HIV DRMs and their exact frequencies are required. Such panels may serve the needs for both EQA and NGS HIVDR assay validation in individual laboratories.
	**Panel specimen types**	▪ Plasma/serum or dried blood spot specimens consisting of - Donor specimens. - Clinical isolates. - Viruses generated from infectious molecular clones.	▪ Plasma/serum or dried blood spot specimens consisting of: - All those listed for SS methods. - Pedigreed plasmids and plasmid mixtures that help for the assessment of the gross error rate and the MRV detection sensitivity of the assay. * VP specimens should be characterized for the DRMs they contain and their exact frequencies. * Initial VP panels may focus on plasma specimens at viral loads of ≥1,000 copies/mL.
	**Panel characterization strategies**	▪ VL level determination using a validated test ▪ HIVDR tests used: - ViroSeqTM HIV-1. - TruGene^®^ HIV-1 (no longer available).	▪ Same technologies for VL determination. ▪ ViroSeq or other commercially validated HIVDR tests if only AP is concerned. ▪ For VP, predetermination of HIV DRMs present and their exact frequencies using unique molecular identifiers (UMI) or other technologies that accurately resolve the abundance of HIV DRMs at a full range of frequencies is required.
	**Panel size**	▪ Five specimens per panel.	▪ 5~10 specimens per panel may be required if aiming to cover HIV DRMs at frequencies <20%. ▪ For logistical and practical reasons, assessing different assay capacities or potentials in accommodating diverse specimens with alternate panels is advised to avoid single large panels.
	**Panel distribution**	▪ Biannual distribution for NIAID supported laboratories; ▪ Biannual or annual distribution for WHO HIVResNet laboratories of different categories.	▪ More frequent panel distributions rather than larger panels may be beneficial for timely identification of issues and remedial actions ▪ A biannual distribution could be a good start considering the high NGS costs to the laboratories.
	**HIV gene targets**	▪ PR, RT, and IN	▪ Same as SS
	**HIV DRM MRV frequency range**	▪ Not applicable.	▪ AP panels may initially focus on DRMs at frequencies of ~20%, while a small number of challenging VP samples with DRMs at 5~15% abundance may also be included. ▪ For VP panels, strategies validating the input HIV template numbers are advised when characterizing the exact frequencies of HIV DRMs. ▪ Statistical analysis may be required to determine acceptable ranges of MRVs at very low frequencies, as VL levels vary.
	**Different VLs/mutation loads**	▪ VL in low to medium range. ▪ Replicates of the same sample diluted to different VL levels may sometimes be included.	▪ Inclusion of ≥1 specimen at low VL (~1,000 copies/mL) is advised regardless of the abundance of target DRMs. ▪ The inclusion of replicates of the same sample diluted to different VLs within one panel and across panels is highly recommended.
**Reference Materials prep and distribution: Dry Panels** [25]	**Genuine raw sequencing data**	▪ Not included in VQA panels; limited experience in WHO HIVResNet ▪ Derived from PT and/or clinical specimens. ▪ Of different qualities. ▪ From commercial and in-house assays.	▪ Derived from PT and/or clinical specimens. ▪ Of different qualities. ▪ Using both commercial and in-house assays. ▪ From different NGS platforms.
	**Synthetic/in silico datasets**	▪ Not included	Such data may complement datasets derived from real specimens for covering: - Different NGS platforms. - Different DRMs, including uncommon ones (e.g., indels). - “Artificial” contamination reads. - “Artificial” incorporation of sequence quality diversity. - Covering PR, RT, and IN. - Specific HIV DRM targets at exact known frequencies.
	**Panel size**	▪ Data files of a different quality from ~100 specimens.	▪ The size of such dry panels could be flexible. ▪ Any data that highlight potential quality assurance (QA) issues and contributes to NGS HIVDR data processing pipeline validation and refinement could be incorporated. ▪ Proper categorization (based on QA issues) and annotation of the files would be required.
	**Data access**	▪ Restricted to HIVResNet designated and candidate laboratories (to date)	▪ Open access through the public domain is recommended.
	**Potential application**	▪ Such panels involve no sample processing in the laboratory and may serve the needs for: - Assessing laboratory capacity for data analysis. - Technical training for appropriate SS HIVDR data management.	Such panels involve no sample processing in the laboratory and may serve the needs for - Assessing laboratory capacity for data processing. - Technical training for appropriate NGS HIVDR data management. - Validation and/or refinement of NGS HIVDR pipelines for accommodating uncommon HIV DRMs and/or NGS data of different quality.
**Data Collection**	**Data submission requirement**	▪ Standardized submission protocol regarding: - What data to submit? - In what format? - How to submit?	▪ Same as for SS * A standardized data collection protocol is essential for streamlining data management and automating subsequent data assessment steps. It is applicable for both SS and NGS methods
	**Consensus sequence**	▪ Yes, in .fasta format	▪ Consensus sequence(s) (in .fasta format) at defined threshold(s) (e.g., 20%) to approximate SS reads for downstream EQA data assessment using current SS strategies.
	**HIV DRM / Variation reports**	▪ Qualitative reports on HIV DRMs present in the specimens.	▪ HIVDR interpretation and reporting based on consensus at a defined threshold using Stanford HIVdb, REGA, ANRS, or other established algorithms for SS-like data assessment [26]. ▪ Qualitative HIVDR interpretation and reporting for all DRMs when their frequencies are >=5% or >=15% [27]. ▪ A comprehensive AAVF report covering all detected amino acid variations (DRMs or non-DRMs) and frequencies is recommended for cross-pipeline and inter-laboratory comparisons [27].
	**Raw sequencing data**	▪ Except for data from the ViroSeq assay, original raw SS files are not collected.	▪ The collection of anonymized raw NGS data (in Fastq format) is encouraged for potential data validation, troubleshooting, or cross-pipeline comparison purposes.
	**Information on the protocols applied**	▪ Categorical protocol information only (in-house, ViroSeq, or TruGene), with minimal laboratory protocol details collected. ▪ Data analysis (base-calling) and reporting software.	▪ Collection of laboratory protocol and data analysis pipeline information is highly recommended for potential data validation, troubleshooting, or cross-pipeline comparison purposes. ▪ A standardized documentation template with all required protocol items (e.g., HIV RNA/DNA extraction, PCR amplification procedures, NGS library preparation kits, NGS sequencing kits, NGS platform, data analysis pipeline, and HIVDR interpretation algorithms) should be applied. ▪ Differences in sample processing and data analysis protocols should be considered for EQA.
	**Data collection approach**	▪ VQA data submission portal	▪ Data submission portal for small-size files, i.e., consensus sequences, AAVF and DR reports. ▪ Cloud sharing of coded raw NGS data files of larger sizes (i.e., Fastq) would be beneficial.
**Data Assessment**	**Guidelines/SOPs**	▪ Well-established SOPs [8,24].	▪ Well-defined EQA data assessment guidelines remain to be established [23]; ▪ EQA assessment based on NGS consensus sequences may oversimplify the complexity of NGS HIVDR data, which detect HIV DRMs both qualitatively and quantitatively.
	**Data assessment parameters** [24]	▪ Concordance with consensus from the group (mismatch counts in the examined HIV genomic regions for each sample and target gene regions). ▪ Error counts in identifying amino acid changes at DRM codons. ▪ Scoring based on established criteria [8,24].	▪ A two-part assessment is recommended while transitioning from SS to NGS HIVDR testing: Part 1: Simplified data assessment using NGS consensus at a threshold of 20% following the current VQA strategies [24]. Part 2: In-depth, NGS-specific EQA data assessment [21,23] ▪ AAVF files containing all amino acid variations, instead of DRMs only, at a wide range of frequencies, may be more informative for cross-laboratory comparisons. ▪ Focus more on the ability to detect DRMs over the desired threshold (e.g., 5%) for scoring, while the accuracy of the DRM frequency readouts should also be assessed.
	**Scoring strategies**	▪ Proficiency scores are based on the number of disagreements from the consensus sequence. ▪ Performance is assessed by assigning a p-value to the observed number of disagreements in a data set for each sample and gene region. ▪ Performance Scores and certifying criteria are well established [24].	▪ Traditional EQA parameters (sensitivity, specificity, linear range, etc.) may not be directly applicable for NGS HIVDR assays due to the uncertain thresholds for MRV detection. ▪ New or redefined parameters are required and meaningful reference, target values, or acceptable ranges for such parameters need to be better defined for NGS HIVDR data assessment and scoring [22,23]. ▪ Proper strategies for identifying “outlier” laboratories and scoring the inconsistencies in DRM detection and frequency readouts among the laboratories have yet to be better defined. [21,23] ▪ Weighting strategies based on the two-part assessment process should be developed for the final laboratory scoring.
**Assessment Reporting**	**Files & contents**	Report files: ▪ Sequence alignment showing consensus sequence and the sequences from all participating laboratories. ▪ Homology report comparing sequences from individual laboratories against the consensus sequence derived from the group. ▪ HIVDR mutation output against the current IAS-USA DRM list (e.g., 2017 [28]). ▪ Scoring/ranking sheet with an explanation of detailed scoring criteria.	▪ A two-part assessment report is recommended: Part 1: A simplified assessment report based on NGS consensus at a threshold of 20% following existing SS strategies. Part 2: In-depth data assessment based on AAVF files and DRM reports collected from participating laboratories against the newly-defined, NGS-specific assessment and scoring criteria: - Spreadsheets, statistical analysis, and graphs showing the performance of the laboratory as compared to its peers. - When required, certification recommendations should be provided. - Issues identified and potential corrective or remedial action recommendations should be provided when possible.
	**Assessment, data distribution and retention/archival**	▪ The assessment reports are emailed to the focal contacts of each laboratory. ▪ VQA retains the assessment documents for the life of the program contract.	▪ The assessment data should be reported back to the focal contacts of the laboratories via email or the data submission portal. ▪ The assessment documents may be retained for the life of the EQA program for subsequent data re-evaluation or validation purposes. ▪ The original NGS data collected from the laboratories may be retained a determined amount of time (e.g., up to 6 months) for cross-checking and re-examination purposes. ▪ Data retention policies from a specific bioinformatics pipeline may also apply. ▪ Guidelines for responsible data sharing among relevant stakeholders should be established.
**Other Challenges**	**Incentives for participation**	▪ Required participation for NIH-funded research programs or projects. ▪ A requirement for WHO HIVResNet laboratory network designation [8]. ▪ Follow-up services such as troubleshooting, corrective action recommendations, and technical training.	▪ A requirement for laboratories wishing to transition to NGS for HIVDR testing and maintain their status in existing networks (e.g., WHO HIVResNet or NIH ACTG). ▪ Allowing voluntary self-paid participation would help to involve more laboratories not belonging to any of the above categories. ▪ Follow-up services for client laboratories, such as technical training opportunities and troubleshooting assistance.
	**Program sustainability**	▪ Continued NIAID funding support. ▪ Availability of experienced technical and administrative staff. ▪ Availability of PT panels that meet the EQA needs. ▪ Affordability of SS assays allowing broader adoption by client laboratories. ▪ Generalized SS HIVDR application in research, surveillance, and clinical settings.	▪ Current challenges for a sustainable EQA program for NGS HIVDR testing laboratories: - Lack of sustainable funding support. - Shortage of experienced technical and administrative staff. - Lack of reference materials or panels suitable for NGS HIVDR EQA applications. - High costs of NGS instruments and consumables despite the gradually dropping prices. - Lack of understanding and appreciation for the potential clinical relevance of MRVs. - Lack of NGS HIVDR data processing tools with unified data processing strategies. - Increasing but still limited adoption of NGS HIVDR assays in the laboratories. - Limited access to technical support for NGS sequencing and data processing
	**Strategies to facilitate SS to NGS transitioning**	▪ Not Applicable	▪ Two-step wet panel (AP and VP) development approach. ▪ Two-part EQA data assessment. ▪ Professional bioinformatics support for unified data processing. ▪ No restriction for NGS platforms and bioinformatics pipeline to be applied by the laboratories.

**Abbreviations** (*in alphabetical order*): **AAVF**: Amino Acid Variation File; **ACTG**: the AIDS Clinical Trials Group; **ANRS**: the HIV genotypic interpretation system from the Agence Nationale de Recherches Sur le SIDA), France; **AP**: Assessment Panel; **CLSI**: Clinical and Laboratory Standards Institute; **DNA**: Deoxyribonucleic Acid; **DRM**: Drug Resistance Mutation; **EQA**: External Quality Assessment; **HIV**: Human Immunodeficiency Virus; **HIVdb**: the HIV resistance interpretation system from the HIV Drug Resistance Database, Stanford University, The United States; **HIVDR**: HIV Drug Resistance testing; **HIVResNet**: Global HIV Drug Resistance Network; **IAS-USA**: the International Antiviral Society-USA; **IN**: Integrase; **MRV**: Minority Resistance Variant; **NGS**: Next-Generation Sequencing; **NIAID**: National Institute of Allergy and Infectious Diseases, the United States; **NIH**: National Health Institutes, the United States; **PAHO**: Pan America Health Organization; **PHAC**: Public Health Agency of Canada; **PR**: Protease; **PT**: Proficiency Test; **QA**: Quality Assurance; **REGA**: the HIV genotypic interpretation system from Rega Institute for Medical Research, Belgium; **RM**: Reference materials; **RNA**: Ribonucleic Acid; **RT**: Reverse Transcriptase; **SOP**: Standard Operating Procedure; **SS**: Sanger sequencing; **UMI**: Unique molecular identifier; **VL**: Viral load; **VP**: Validation Panel; **VQA**: Virology Quality Assurance program supported by NIAID; **WHO**: World Health Organization.
